# Influence of Critical Shoulder Angle and Rotator Cuff Tear Type on Load-Induced Glenohumeral Biomechanics: A Sawbone Simulator Study

**DOI:** 10.1155/2024/4624007

**Published:** 2024-07-02

**Authors:** Jeremy Genter, Eleonora Croci, Andreas M. Müller, Annegret Mündermann, Daniel Baumgartner

**Affiliations:** ^1^ IMES Institute of Mechanical Systems Zurich University of Applied Sciences ZHAW, Winterthur, Switzerland; ^2^ Department of Biomedical Engineering University of Basel, Basel, Switzerland; ^3^ Department of Orthopaedics and Traumatology University Hospital Basel, Basel, Switzerland; ^4^ Department of Clinical Research University of Basel, Basel, Switzerland

## Abstract

Glenohumeral (GH) biomechanics after rotator cuff (RC) tears are not fully understood. The purpose of our study was to determine if the critical shoulder angle (CSA), type of RC tears, and level of weight bearing increase GH translation, instability based on the instability ratio, muscle forces and joint reaction force (JRF), and shifts the center of force (CoF) superiorly. A GH simulator with muscle-mimicking cable systems was used to simulate 30° abduction in the scapular plane. A Sawbone humerus and five specimen-specific scapular anthropometries were used to test six types of RC tears, three weight-bearing loads, and the native and adjusted (to different CSAs) deltoid origin sites. Linear mixed effects models (CSA, RC tear type, and weight bearing) with random effects (specimen and sex) were used to assess differences in GH biomechanics. With increasing CSA, GH translation increased, JRF decreased, and the CoF position was more inferior. RC tears did not significantly alter GH translation but shifted the CoF position superiorly, close to where glenoid erosion occurs in patients with RC tears with secondary osteoarthritis. Weight bearing significantly increased GH translation and JRF. RC and deltoid muscle forces increased with the presence of RC tears and increased weight bearing. The remaining RC muscles of intact tendons compensated for the torn RC tendons but not for the altered CoF position. GH translation remained comparable to shoulders with intact RC. These findings highlight the importance of early detection, clinical management, and targeted rehabilitation strategies for patients with RC tears.

## 1. Introduction

The glenohumeral (GH) joint has the largest range of motion (RoM) of any human synovial joint, due in part to the size discrepancy between the small glenoid and the prominent humeral condyle. The GH joint has six degree of freedom (DoF), three rotational and three translational, and is controlled by 11 muscles [1]. During abduction, the rotator cuff (RC) provides stability to the GH joint by limiting inferosuperior translation to a range of a few millimeters [2]. In addition to their stabilizing role, the RC muscles are directly involved in shoulder movement. For example, the supraspinatus (SSP) assists in GH abduction, while the infraspinatus (ISP) and teres minor (TMin) facilitate external rotation and the subscapularis (SSC) facilitates internal rotation. In particular, the SSP shows its highest activity during the early phase of GH abduction [3]. The concavity-compression mechanism involves shear forces, compressive forces, and glenoid shape and contributes to shoulder stability. Shear forces cause inferosuperior instability, while the RC muscles provide compressive forces for stability [4], which may be compromised in shoulders with RC tears.

Anatomical factors such as the critical shoulder angle (CSA) are radiological parameters often assessed as part of clinical routine because a large CSA (>35°) is a risk factor for RC tears and may contribute to GH joint instability after RC tears [5]. The line of action of the deltoid (DELT) muscle is directed more superiorly as its origin at the acromion expands laterally with a larger CSA, resulting in increased GH shear forces, especially during abduction when the DELT is primarily activated [5, 6]. In addition, GH instability and translation are most pronounced at small abduction angles due to the superior translational force of the DELT muscle [2]. For this reason, acromioplasty is gaining popularity to reduce lateral acromial extension thereby reducing the CSA [7, 8]. Vierhöfer et al. [9] suggested in their finite element simulation study with intact RC muscles that as CSA increases, the muscle activity of RC muscles increases to maintain similar levels of GH stability and translation, yet they did not investigate if and how different RC tear types may affect instability and translation at different CSAs.

While Millett et al. [10] reported inferior GH translation in patients with RC tears (full thickness; size >1 cm) compared to healthy individuals (significant only at higher abduction angles), Kozono et al. [2] reported increased superior GH translation in patients with RC tears (full thickness; size >3 cm), especially at small abduction angles (not significant). The CSA, size and location of the RC tears may have contributed to this discrepancy. Overall, the current understanding of the factors underlying differences in GH biomechanics in patients with RC tears remains incomplete. While in vivo studies provide physiological data from patients, in-depth analysis of GH biomechanics is limited by the challenges associated with measuring muscle and joint reaction forces (JRFs) to determine joint stability.

Ex vivo approaches offer the potential to measure these variables but are methodologically limited by the complex task of replicating in vivo behavior. Using GH simulators, Kedgley et al. [11, 12] found inferior GH translation with RC tears compared to intact shoulders, while other studies [13, 14, 15] reported superior GH translation with inactive RC muscles. Differences in experimental simulators may contribute to these discrepancies due to differences in muscle force application. Kedgley et al.'s [11, 12] simulator used a predetermined muscle force ratio based on healthy electromyography (EMG) patterns, whereas other simulators mimicked isotonic muscle contractions of RC muscles and controlled the DELT muscle force [13, 15, 16, 17] or used uniformly distributed muscle forces that were determined iteratively [14]. Expanding on current GH simulators summarized in a recent scoping review [18], we developed a GH simulator that employs a musculoskeletal model-based control strategy that accounts for GH stability [19].

In this study, we used this custom-built GH simulator to replicate isolated full-thickness RC tears and massive tear types A, C, and D of Collin et al.'s [20] classification on a Sawbone model on the initiation of the abduction movement up to 30° in the scapular plane. The purpose of our study was to determine if the CSA, type of RC tears, and level of weight bearing increase GH translation, instability based on the instability ratio (IR), muscle forces and JRF, and shifts the center of force (CoF) superiorly.

## 2. Materials and Methods

### 2.1. Testing Apparatus

**Figure 1:**
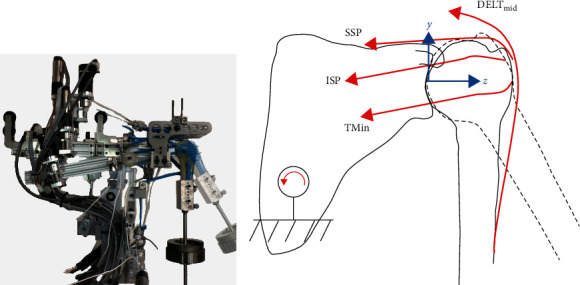
(a) Photograph of the simulator. The simulator arm position is shown in the neutral position (opaque) and in 30° abduction (transparent). (b) Schematic of the muscle forces acting—the anterior and posterior portions of the deltoid, subscapularis, latissimus dorsi, and the pectoralis major muscles are not shown for simplicity. The position of the simulator arm is shown in neutral position (solid line) and in 30° abduction (dashed line). The glenoid coordinate system is shown in blue. DELT_ant_ – anterior portion of the deltoid muscle; SSP – supraspinatus muscle; ISP – infraspinatus muscle; and TMin – teres minor muscle.

To evaluate the GH biomechanics, we used our custom-designed GH simulator [19] with a free-hanging humerus (Figure 1). In a previous study [19], we showed the simulator's ability to consistently achieve 30° of GH abduction, with median errors ranging from 0.7° to 1.4°. In addition, we validated that the JRF at 30° abduction and different weight-bearing levels closely matched in vivo measurements [21]. Our simulator consists of 10 actuated muscle units, three for the DELT muscle (anterior (DELT_ant_), middle (DELT_mid_), and posterior (DELT_post_)), two for the SSC muscle (superior (SSC_sup_) and inferior (SSC_inf_)), and one each for the SSP, ISP, pectoralis major (PECMaj), and latissimus dorsi (LAT) muscles, as well as a simulated arm (right humerus, solid foam, Sawbones, Washington, USA). The muscles are actuated by cable systems attached to electromotors. The insertion sites of the muscles are glued to the corresponding anthropometry on the humerus and additionally fixed with screws. The origins at the scapula are mimicked with adjustable cable pulleys, allowing the cables to effectively wrap around the humerus. The simulator is controlled in a cascade layer for joint position (measured with an inertial measurement unit; Tinkerforge, Schloß Holte-Stukenbrock, Germany) and muscle forces. The joint position cascade provides input to a real-time optimizer that determines muscle forces, while maintaining a stable concavity-compression mechanism as described in our previous work [19]. In short, the real-time optimizer takes into account the pre-estimates from the musculoskeletal model [19] using the muscle force optimization algorithm by Wu et al. [22]. They developed muscle force optimization with the constraint of keeping the resulting JRF within the glenoid to maintain a stable concavity compression mechanism while minimizing the sum of the squared muscle activity. The muscle force cascade layer ensures that the muscle forces determined by the optimizer are achieved.

### 2.2. Sawbone Specimen Preparation

Sawbone specimens were used to replicate five freshly frozen cadaveric shoulders anthropometries (two females, three males) which were obtained as part of a larger project approved by the Zurich Ethics Committee (BASEC No. 2022-00460) [23]. Specifically, the arm was simulated by a right proximal Sawbone humerus with an anatomical implant (humeral head diameter: 44 mm; Plus, Aarau, Switzerland) and a custom-made polyethylene glenoid prosthetic component (curvature diameter: 60 mm; Mathys, Bettlach, Switzerland), and the arm weight was simulated by attaching a mass of 2.8 and 3.1 kg at 29 and 30 cm distal to the center of the GH joint for female and male specimens, respectively [24]. The glenoid component was embedded in an adapter laterally to a 6-DoF force sensor (Transmetra, Schlattingen, Switzerland). To personalize the simulator setup for each individual specimen, the origin sites of the muscle portions of the DELT, PECMaj, and LAT on the scapula were adjusted based on specimen-specific anthropometry obtained from computed tomography scans of the fresh-frozen cadaveric shoulders. To evaluate the effect of acromial lateralization, as measured by the CSA, on the biomechanical outcomes, we tested each specimen twice; once with the specimen's native CSA and once with a modified CSA. Depending on the native CSA (closer to healthy (32°) or closer to the prevalent CSA in patients with RC tears (37°)) [5], the site of origin of the DELT_mid_ was adjusted to match the opposite CSA for each specimen, resulting in 10 test anthropometries.

### 2.3. Experimental Protocol

We simulated all Sawbone shoulders with intact RC and six types of RC tears: SSP, SSC_sup_, ISP, SSP + SSC_sup_ (Collins type A), SSP + ISP (Collins type D), and SSP + SSC_sup_ + ISP (Collins type C; in that order). During the experiment, no force was applied to the simulated ruptured RC tendons, which simulated a full-thickness and full-width tear of the corresponding simulated tendon attachments. For each RC condition, we tested a 30° GH abduction–adduction cycle in 6 s in the scapular plane with a neutral internal rotation of the humerus and simulated three different weight-bearing loads (0, 1, and 2 kg) by attaching weights to the distal end of the arm (arm length, female: 71 cm; male: 74 cm) [24]. Each of the 21 conditions was repeated three times for each Sawbone specimen, and the mean of the three trials was calculated for each Sawbone specimen and condition.

### 2.4. Data Acquisition

**Figure 2:**
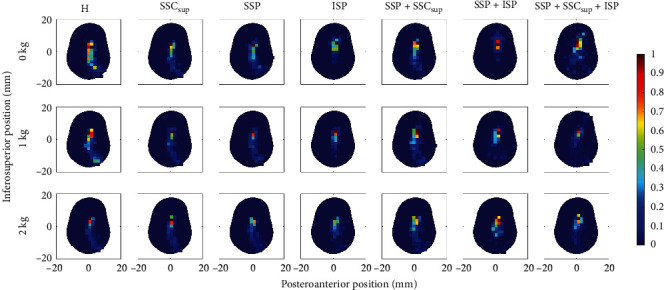
Spatial histogram of the center of force (CoF) position throughout the abduction movement and additional loads (0, 1, and 2 kg). The colors represent the number of crossings of the CoF in a given region throughout the movement. The number of crossings was normalized to the maximum number of crossings of the regions with the maximum crossing. H – intact rotator cuff (RC); SSC_sup_ – RC tear of superior portion of the subscapularis muscle; SSP – RC tear of supraspinatus muscle; ISP – RC tear of infraspinatus muscle.

A marker-based motion capture system (OptiTrack, Corvallis, Oregon, USA; 100 Hz; calibration error: 0.05 mm) was used to track the 6-DoF kinematics of the joint. The instantaneous helical axis method [25] was used to identify the GH joint center in the humerus-fixed coordinate system. The movement with the intact RC was used as a reference, and the best-fit intersection of all instantaneous helical axes during the movement was defined as the joint center. We then tracked the inferosuperior position of this point and measured the GH translation from the start of the movement to the 30° abduction in the glenoid-fixed coordinate system (origin: centroid of the glenoid rim shape obtained from the computer-aided design (CAD) of the glenoid component; *X*-axis: anterior (+)-posterior (−); *Y*-axis: superior (+)-inferior (−); *Z*-axis: lateral (+)-medial (−); Figure 1(b)). JRF was measured directly behind (medially of) the polyethylene glenoid component using the 6-DoF force sensor (accuracy: 1%). The IR [26], which describes the concavity-compression mechanism, was calculated as follows:

(1) $$\begin{array}{c} \text{IR}=\frac{\left|f_{\text{shear}}\right|}{\left|f_{\text{comp}}\right|}, \end{array}$$

where **f**_shear_ and **f**_comp_ are the GH shear and compression forces. The CoF was calculated as the intersection of the force line (from the 6-DoF force sensor) and the best-fit sphere of the glenoid (from the CAD model). We visualized the CoF position throughout the movement using a 2D histogram on the lateral view of the glenoid (Figure 2). The lateral view of the glenoid was divided into a grid of pixels (pixel size: 1.75 × 1.75 mm), and we tracked which pixels the CoF crossed during movement. By aggregating the data from all specimens, we determined the frequency of CoF crossings for each pixel, which represents the CoF distribution. For statistical purposes, the inferiosuperior position of the CoF was tracked in the glenoid-fixed coordinate system. The amplitude of the muscle forces was measured by individual force sensors for each simulated muscle (Interfaceforce, Tegernsee, Germany; accuracy: 0.15%). For all outcomes (GH translation, IR, CoF, and JRF in the GH joint and muscle forces), we calculated the value at 30° abduction and the time-averaged value to evaluate all outcomes throughout the movement.

To calculate the time-averaged value, we integrated the result over the entire 30° abduction–adduction cycle time and then divided it by the total duration of the movement. This approach provided an overall representation of the outcome over the entire movement cycle. By comparing the time-averaged value to the value at 30° abduction, potentially nonlinear trajectories can be identified (i.e., a notable difference between the two values). Due to some outliers of the IR during the start and end of the movement, we did not analyze the time-averaged IR. During these phases, the shear forces in the GH joint increased greatly while the compressive forces remained relatively low, resulting in a large increase in the IR. Thus, the IR indicates instability only when the humeral head is in a decentering phase: When the humeral head center is inferior to the glenoid center and the shear forces are directed upward, the shear forces will centralize the humeral head, but when the humeral head is already superior to the glenoid center and the shear forces are directed upward, then the shear forces will decenter the humeral head even more, resulting in instability when the shear forces exceed a certain limit. However, we did not observe any decentering of the humerus. The analysis was performed in MATLAB 2020a (The Mathworks, Natick, MA, USA).

### 2.5. Statistical Analysis

We examined whether different types of RC tears, weight-bearing load, and CSA contribute to increases in inferiosuperior GH translation, IR, muscle forces and JRF, and shifts in the CoF position using a linear mixed-effects model. As a first step, we performed a stepwise linear regression model to identify the variables that explained the most variance in each GH biomechanical outcome (i.e., inferiosuperior GH translation, IR, muscle forces, JRF, and shift in the CoF position) at 30° abduction and for the time average. It should be noted that for the IR, we only performed the statistics at 30° abduction. The initial predictor variables were CSA, RC tear type, and weight bearing. The RC tear types were treated as categorical variable and each tear type was represented by a dummy variable, while the other predictor variables were treated as continuous variables. This approach allowed us to systematically evaluate the predictor variables and identify the subset of predictor variables that were most strongly correlated with the outcomes. A linear mixed effects model was then used to assess the significance of the most strongly correlated preselected predictor variables. While this method allowed for systematic evaluation and identification of strongly correlated predictors, we did not explore interactions between these predictors due to our limited number of anthropometries. To account for differences between specimens, such as muscle origin sites, arm length, and arm weight, the uncorrelated random effect intercept was grouped by specimen and sex, while the uncorrelated random effect of weight bearing was grouped by sex. The significance level was set at 0.05 and the analysis was performed in MATLAB 2020a.

## 3. Results

The median of the native and adjusted CSA of the specimens tested was 33.7° (range, 28.4°–45.0°). The actual mean (standard deviation) of the achieved abduction angle was 30.8° (1.3°). The stepwise linear regression model identified CSA and weight-bearing load as fixed effect predictor variables for the linear mixed effects model of the GH translation. The RC tear types were identified as additional fixed effect predictor variables for the outcome variables: CoF, the magnitude of JRF, and muscle forces. The stepwise linear regression of the IR considered RC tear type and CSA as fixed effect predictor variables. Descriptive statistics for the outcome variables for all load and tear type conditions are presented in Supplementary Material (available here) (Zenodo: [27]), and the mean trajectory ± standard deviation of the measurements is presented in the supplement.

### 3.1. Critical Shoulder Angle

**Figure 3:**
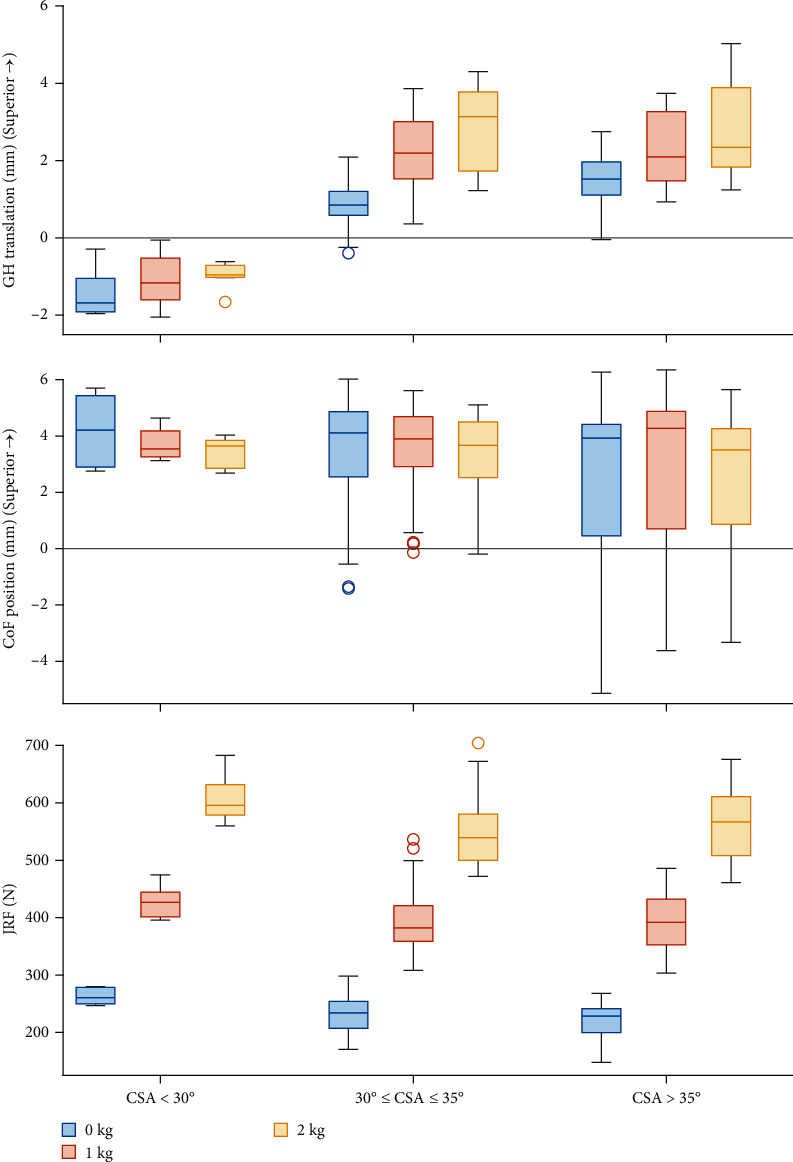
Box plots of glenohumeral (GH) superior translation, center of force (CoF) superior translation, and joint reaction forces (JRF) grouped by small, healthy, and large critical shoulder angle (CSA) (according to Moor et al. [5]) for visualization purposes only, and weight-bearing loads (0, 1, and 2 kg).

A greater CSA was associated with a more inferior position of the CoF and a superior GH translation at 30° abduction (*p* < 0.001) and throughout the movement (time-averaged; *p* ≤ 0.007). The JRF decreased with increasing CSA at 30° abduction (*p* < 0.001) and throughout the movement (*p* < 0.001; Figure 3). The CSA did not affect the IR at 30° abduction (*p*=0.134; Table 1). Regarding muscle forces, the DELT_mid_ force increased slightly with increasing CSA at 30° abduction (*p*=0.028) and throughout the movement (*p*  < 0.001). None of the RC muscle forces at 30° abduction were associated with a change in the CSA (*p* ≥ 0.184; Tables 2 and 3).

### 3.2. Rotator Cuff Tear

**Figure 4:**
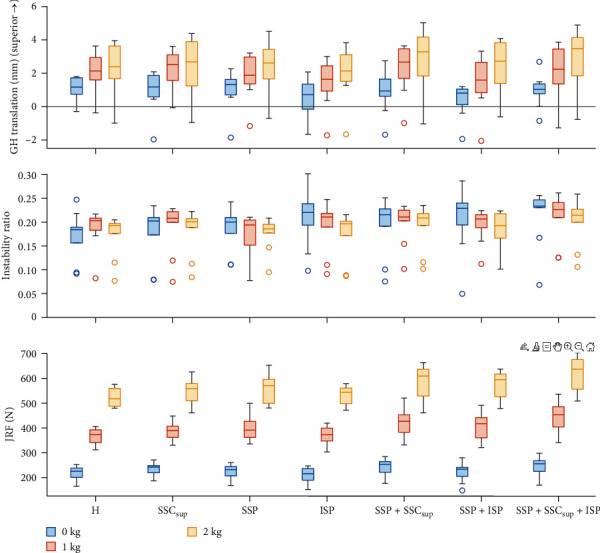
Box plots of the glenohumeral (GH) superior translation, instability ratio (IR), and joint reaction forces (JRF) at 30° abduction grouped by rotator cuff (RC) tear type and weight bearing loads (0, 1, and 2 kg). H – intact rotator cuff (RC); SSC_sup_ – RC tear of superior portion of the subscapularis muscle; SSP – RC tear of supraspinatus muscle; ISP – RC tear of infraspinatus muscle.

**Figure 5:**
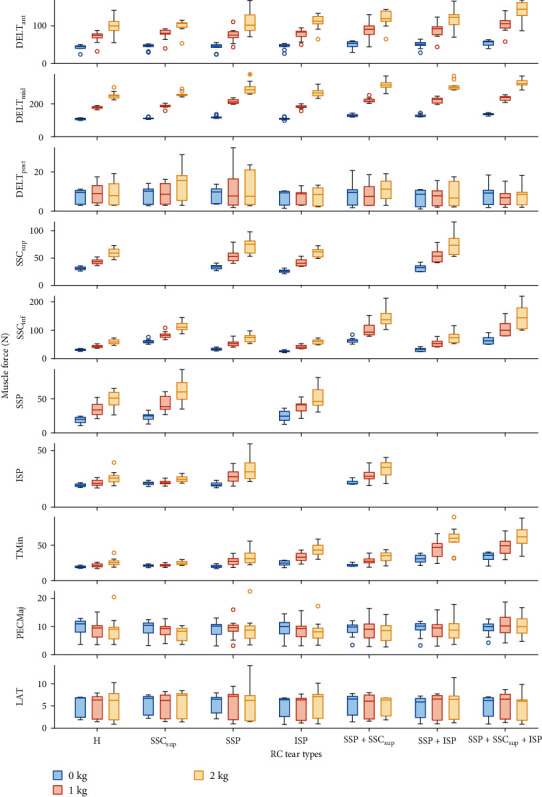
Box plot of the muscle forces at 30° abduction grouped by rotator cuff (RC) tear type and weight-bearing loads (0, 1, and 2 kg). H – intact RC; DELT_ant_ – anterior deltoid portion; DELT_mid_ – middle deltoid portion; DELT_post_ – posterior deltoid portion; SSP – supraspinatus; SSC_sup_ – superior subscapularis portion; SSCinf – inferior subscapularis; ISP – infraspinatus; TMin – teres minor; PECMaj – pectoralis major; LAT – latissimus dorsi.

The distribution of the CoF position throughout the abduction movement of all specimens is shown in Figure 2. In intact RCs, the CoF was mostly positioned along the inferosuperior glenoid axis. For RC tear types A, C, and D and the ISP tear the CoF was displaced superiorly at 30° abduction (*p* < 0.001) and throughout the movement (*p* < 0.001; Figure 2) compared to the intact RCs. For the SSP tear additionally, the CoF was displaced superiorly throughout the movement (*p*=0.038). At 30° abduction, the RC tear types A, C, and D and the ISP tear increased the IR at 30° abduction (*p* ≤ 0.011). The magnitude of the JRF increased with all RC tear types (*p* ≤ 0.039) except the ISP tear (*p* ≥ 0.175) at 30° abduction and throughout the movement. The GH translations were not affected by RC tears (Figure 4; Table 1). The DELT_ant_ and DELT_mid_ forces increased with the RC tear types A, C, and D (*p* < 0.001) at 30° abduction. The intact anterior RC compensated for anterior tears, and the intact posterior RC compensated for posterior tears throughout the movement and 30° abduction, reflected in greater required muscle force (*p* < 0.001). The SSP force increased with SSC_sup_ tear (*p* < 0.001) but not with ISP tear (*p*=0.056). Throughout the movement, all muscle forces were affected by most RC tear types (*p* ≤ 0.038) except for the LAT force (*p* ≥ 0.173; Figure 5; Tables 2 and 3).

### 3.3. Weight Bearing

An increase in weight-bearing load increased GH translations and JRF at 30° abduction (*p* ≤ 0.018) and throughout the movement (*p* ≤ 0.001; Figures 3 and 4). An increase in weight-bearing load was significantly associated with a more inferior CoF position throughout the movement (*p* < 0.001), but not at 30° abduction (*p*=0.643). The IR was not affected by the difference in weight-bearing load (Figure 2; Table 1). Muscle forces increased with increasing weight-bearing load at 30° abduction (*p* < 0.001) and throughout the movement (*p* < 0.001) except for the DELT_post_, PECMaj and LAT (*p* ≥ 0.198; Figure 5; Tables 2 and 3).

## 4. Discussion

Our simulator Sawbone experiments provided important insights into the contribution of predictor variables and their differential effects on biomechanical outcome variables. Specifically, anatomical variation, as measured by CSA, had a predominant effect on GH translation, and RC tears significantly influenced CoF, IR, and muscle forces. In addition, the level of weight bearing significantly increased GH translation, JRF, and muscle forces. Thus, each predictor variable had a distinct effect on the outcome variables in our study.

GH translations increased with increasing CSA at 30° abduction and throughout the abduction and adduction movement. This finding is consistent with a previous ex vivo study by Bouaicha et al. [28], who also reported increased GH translation with increasing CSA. Clinicians often observe humeral head migration on diagnostic radiographs in patients with massive RC tears, and Verhaegen et al. [29] also observed that a greater CSA increased humeral head migration in patients with massive RC tears. The increased GH translation in our study supports this finding. Interestingly, increasing CSA minimally increased DELT_mid_ forces while RC muscle forces at 30° abduction remained unaffected. Nyffeler et al. [6] proposed that the lateralization of the acromion favors impingement and degeneration of the SSP muscle because the DELT_mid_ force vector is directed more superiorly, which is supported by our findings of increased GH translation. Given the marginally increased forces in the DELT_mid_, this resulted in a minimal nonsignificant decrease in the forces exerted by the remaining DELT portions and RC muscles. The sum of these decreased forces then resulted in a slightly decreased JRF. However, in contrast to our results, a finite element analysis study (Viehöfer et al. [9]) found that increasing the CSA affected the IR. The discrepancy in the results may be due to the different optimization approaches. Viehöfer et al. [9] solved for muscle forces by minimizing the sum of the muscle stresses and optionally adding RC muscle forces, whereas our optimization in the simulator solved directly for muscle stress minimization and joint stability, which may explain the differences in our results. Finally, we observed that increasing the CSA had only a marginal effect on the JRF and no effect on the IR suggesting that the CSA may not have a clinically relevant effect on the JRF. In clinical practice, the CSA is a valuable tool for treatment evaluation. Recent studies [7, 8] have investigated acromioplasty as a treatment to reduce the CSA. Our study confirms that reducing acromial lateral extension, and thereby reducing CSA, may be an effective way to minimize GH translations.

In the presence of RC tears, the DELT_ant_ and DELT_mid_ and the remaining intact RC had increased their muscle forces to maintain stability in the joint, and hence we did not find a significant effect of RC tears on GH translation. In addition, it was observed that the intact anterior RC compensated for anterior tears and the intact posterior RC compensated for posterior tears. Specifically, for isolated SSC_sup_ tendon tears and tear types A and C the SSC_inf_ force was increased. Similarly, TMin force was increased for the isolated ISP tendon tear and tear types C and D. In addition, we found increased DELT_mid_ for the isolated SSP tendon tear and increased DELT_mid_ and DELT_ant_ forces for all massive RC tears. Strengthening the muscles that compensate for the specific torn RC tear types may be advisable, as this may increase the specific compensatory capacity to maintain stability in the injured shoulder. Strengthening the RC muscles should be the primary focus, as the proposed mechanism of increased GH translations involves the superior directed pull of the DELT muscles. Therefore, training the RC muscles to stabilize the joint should precede the use of the DELT muscles for compensatory actions in GH abduction, as suggested by the observed compensations. This prioritization ensures that the RC muscles are sufficiently prepared to maintain shoulder stability before the DELT muscles are engaged for compensation. Moreover, most RC tear types significantly affected all muscle forces except the LAT for time-averaged force. Since the PECMaj and DELT_post_ were only affected by the RC tears throughout the movement but not at 30° abduction, it appears that these muscles play a critical role in maintaining the humerus on its trajectory.

Based on the systematic review by Shepet et al. [30], who proposed a new standardized rehabilitation protocol for the nonsurgical treatment of chronic, massive, irreparable RC tears, their recommendations are that physical therapy should first address passive forward flexion and external rotation. Second, strengthening of the DELT and TMin muscles is suggested, and third, scapular stabilization is recommended. Based on our findings and recognizing our limitations regarding RoM and incomplete validation of the muscle optimization algorithm, we propose an expansion of the rehabilitation protocol. We advocate the inclusion of stabilizing exercises involving the remaining intact RC muscles in addition to DELT strengthening. Specifically, we suggest the inclusion of submaximal isometric exercises with a progression to unstable isometric holds, such as the inverted kettlebell carry [31]. While our muscle optimization algorithm aimed to keep the concavity-compression condition stable, the CoF shifted superiorly, especially for massive RC ruptures involving the ISP tendon (types C and D) and the isolated ISP tendon tear, largely due to DELT muscle forces. Thus, we expect that sufficient stabilizing activation of the intact RC muscles may limit this superior shift.

Other important findings were that the 30° abduction and the time-averaged position of the glenoid CoF moved superiorly in the isolated ISP tear and all massive RC tear types. In the nonsubluxated shoulder, it is likely that GH translations are constrained by the congruence between glenoid and humeral condyle diameters. As a result, significant changes in CoF may result in only small changes in translation. However, with sufficient superior erosion, the humeral head may migrate superiorly, which may explain the radiographic diagnosis of some RCs [32]. Contrary to the hypothesis of the radiographic review by Moosikasuwan et al. [33] that the increased DELT forces in shoulders with RC tears are not counteracted and thus result in superior humeral head migration, our results suggest that, mechanically, the remaining RC muscles have the potential to compensate for tears to maintain the humeral head in the glenoid. However, we recognize that we have neglected the potential muscular insufficiency of the intact RC muscles and have assumed that the joint is always stable.

The discrepancy observed in the time-averaged CoF between the torn and the intact RCs may provide valuable information regarding the trajectory of the glenoid CoF (Figure 2) and, consequently, allow the identification of potential sites of glenoid erosion leading to secondary osteoarthritis after RC tears. This discrepancy was most pronounced for massive RC tears involving the ISP tendon and isolated ISP tears. For instance, Ozel et al. [34] observed a more superior glenoid erosion in patients with osteoarthritis and RC tears compared to patients with osteoarthritis alone. Moreover, the presence of massive RC tears increased the IR especially in all massive RC tears, which may also contribute to glenoid erosion because greater shear forces result in a higher IR. Abnormal shear stresses are a risk factor for cartilage degeneration, even more so in already degenerated cartilage [35]. Moreover, as summarized by Sanchez-Adams et al. [36], hyperphysiological loads lead to cartilage matrix apoptosis, which also supports the claim that the abnormal shear stresses may lead to secondary osteoarthritis after RC tear. The increased JRF due to the compensatory response of the muscles (e.g., by 7% for the RC tear type C without weight bearing) may also contribute to the development of secondary osteoarthritis after RC tears.

Recommendations on when to consider reverse shoulder arthroplasty for irreparable massive RC tears have been published by Sellers et al. [37]. In general, older patients with chronic irreparable massive RC tears or pseudoparalysis are recommended for reverse arthroplasty in the absence of arthritis. Caution in the widespread use of RSA in the absence of arthropathy is advocated by Sellers et al. [37]. Based on our results, we suggest that patients with massive RC excluding the ISP tendon (type A) are less suitable for early shoulder arthroplasty because they have a smaller superior shift of the CoF compared to the other massive RC tears. However, this needs to be further investigated.

GH translations and JRF increased mainly with increasing weight-bearing load at 30° abduction and throughout the abduction and adduction movement. In the initial position of the humeral head, the additional weight-bearing load induced a downward pull, which mainly caused the increase in GH translation. In fact, once in the abducted position, the position of the humeral head remained relatively stable across all loads. The increase in DELT_ant_ and DELT_mid_ forces with increasing weight-bearing loads was presumably to achieve the required GH abduction torque with the additional load, while the increase in RC muscle forces may have been necessary to withstand the increased shear forces. Our results on the effect of weight bearing on GH translation emphasize that this aspect is a critical aspect of the study of shoulder biomechanics in health and disease.

The experimental setting with a simulator in a Sawbone study allowed us to study the detailed biomechanics of the shoulder. In particular, we were able to systematically study the effect of CSA, RC tear types, and weight bearing on GH translation, CoF, joint stability, JRF, and muscle forces. Our simulator showed promising results in reproducing the JRF of an in vivo study of instrumented shoulder implants conducted by Bergmann et al. [21], which was also demonstrated in our previous study [19]. Furthermore, the observed GH translations are consistent with other in vivo fluoroscopic findings, with Millett et al. [10] reporting mean (standard deviation) inferior–superior GH translations of 0.9 mm (1.8 mm) for controls and 0.6 mm (0.7 mm) for the RC tear group at 30° GH abduction, and Kozono et al. [2] reported mean (standard deviation) inferior GH translations of 0.8 mm (0.7 mm) for controls and 1.5 mm (1.2 mm) for the RC tear group. Consistent with our study, both studies found no significant differences in GH translations at 30° abduction between the control and RC tear groups. This further supports the design and value of our simulator. However, because infinite combinations of muscle forces can produce similar GH JRFs and translations, validation of this muscle optimization is needed.

Unlike other simulators in the literature, we use an optimization scheme to simulate RC tears while taking GH stability into account. Some simulators use fixed ratio or isotonic forces to determine muscle force, while others incorporate healthy EMG data [18]. While methods based on fixed ratio, isotonic forces, and healthy EMG patterns are simplifications without considering compensatory mechanisms, our approach attempts to achieve more pathologic-specific muscle activation by incorporating an optimization scheme that considers GH stability to accurately mimic movement in shoulders with RC tears. However, it is not clear whether the stability criteria of the concavity-compression mechanism is reflected in in vivo subjects, and it would be beneficial to confirm whether and to what extent this optimization reflects a physiological compensation in an in vivo setting. Our chosen method of muscle force optimization does not take into account the absence or insufficiency of compensatory strategies for joint stability. Therefore, the results should be interpreted as a potentially achievable compensatory mechanism through targeted muscle activation patterns and strength training in patients with RC tears. An in vivo study [38] showed a 59% success rate with physical therapy, suggesting that some patients achieve sufficient muscle compensation. However, our simulator does not clarify why the success rate is only moderate. Moreover, it should be noted that the current study was limited to shoulder abduction angles up to 30°, and further studies with a wider range of daily activities are needed to obtain a complete picture of GH biomechanics. Therefore, the results should only be interpreted for daily-live-activity with low RoM such as carrying groceries. In addition, we assumed that the scapula remained fixed during 30° of GH abduction. This approach does not take into account that rotation of the scapula may help to compensate for RC tears and may cause changes in the load on the compensatory muscles.

In our investigation, we focused only on the effect of acromial lateralization as one aspect of CSA. In our experiments, we simulated a large variability in CSAs as observed in clinical cohorts. However, these anatomical variations were imposed on only five anatomical specimens. The small intracorrelation coefficients reflected limited variability in our results between specimen suggesting that most of the variability can be attributed to the fixed effect predictor variables. For a more complete understanding, it would be valuable to include the assessment of glenoid inclination. Considering both factors together would provide a more complete and more detailed insight into the interactions and potential effects on shoulder biomechanics. Furthermore, an ex vivo study on our simulator would further strengthen our findings, as cadaveric shoulders would exhibit greater variability in glenoid and humeral shape. In addition, native soft tissues such as the GH capsule, which also serves as a passive stabilizer, and the GH tendons could be preserved in an ex vivo experimental setup. In contrast, our study used an anatomical shoulder prosthesis and a Sawbone model, which do not replicate the varying anatomical shapes of condyles and muscle insertion sites on the humerus. Furthermore, our discretization of muscle parts into 10 segments contrasts with the complex composition of the native shoulder muscle, which is composed of numerous muscle fibers originating from larger areas, such as the origin of the DELT muscle, which extends from the distal part of the clavicle to the medial part of the scapular spine. As a result, some muscles are more complex and may be controlled by multiple individual parts, potentially limiting the direct translation of our findings to in vivo conditions. We have partially addressed this limitation by using three individual portions of the DELT, although further investigation may be necessary to fully capture the complexity of the muscle. Nonetheless, our experimental setup provides insight into possible biomechanical causes of joint degeneration in shoulder muscle pathology. Furthermore, our simulator may be used to investigate the biomechanical implications of novel surgical approaches such as those involving morphological changes, as shown in a recent case study by Gerber et al. [39], who demonstrated that osteotomy involving morphological modifications of the acromion and glenoid to correct abnormal morphological parameters can lead to improved patient outcomes.

## 5. Conclusions

Our study demonstrated the contribution of several factors to GH biomechanics. In particular, we found that reducing the CSA, as can be done surgically with acromioplasty, reduces GH translation. Moreover, specific muscles compensate for specific RC tears, which may assist in deciding which specific muscles should be strengthened in physical therapy. The superior shift of the position of the CoF to close to where glenoid erosion occurs in patients with RC tears with secondary osteoarthritis emphasizes the need for early (conservative or surgical) intervention aiming at maintaining or reestablishing GH joint centering. Overall, our findings underscore the importance of early biomechanical assessment, appropriate treatment, and targeted rehabilitation strategies in patients with RC tears.

## Figures and Tables

**Table 1:** Fixed effects estimates [confidence interval] (*p*-values) of the linear mixed effects models for glenohumeral (GH) joint center translation, glenoidal center of force (CoF), instability ratio (IR), and magnitude of the GH joint reaction force (JRF).

Model parameters	Outcome						
	GH translation		Glenoidal CoF		IR	JRF	
	30° abduction	Time averaged	30° abduction	Time averaged	30° abduction	30° abduction	Time averaged
Fixed effect							
Intercept	−3.9 ^*∗*^ [−5.0 to −2.8] (<0.001)	−1.9 ^*∗*^ [−2.4 to −1.4] (<0.001)	6.9^*∗*^ [4.4–9.5] (<0.001)	1.6 [−1.2 to 4.4] (0.250)	0.16^*∗*^ [0.11–0.21] (<0.001)	272.2^*∗*^ [231.7–312.7] (<0.001)	157.9^*∗*^ [126.5–189.3] (<0.001)
Weight-bearing task	0.9^*∗*^ [0.2–1.7] (0.018)	0.6^*∗*^ [0.4–0.9] (<0.001)	−0.1 [−0.4 to 0.2] (0.643)	−1.3 ^*∗*^ [−1.9 to −0.6] (<0.001)	N/A	163.5^*∗*^ [144.5–182.6] (<0.001)	73.3^*∗*^ [61.1–85.6] (<0.001)
CSA	0.1^*∗*^ [0.1–0.2] (<0.001)	0.1^*∗*^ [0.1–0.1] (<0.001)	−0.1 ^*∗*^ [−0.2 to −0.1] (<0.001)	−0.1 ^*∗*^ [−0.2 to 0] (0.007)	0.00 [0–0] (0.134)	−2.1 ^*∗*^ [−2.8 to −1.4] (<0.001)	−1.4^*∗*^ [−1.9 to −0.8] (<0.001)
SSC_sup_	N/A	N/A	0.5 [0–1] (0.06)	0.3 [−0.6 to 1.3] (0.476)	0.01 [0–0.02] (0.195)	20^*∗*^ [9.5–30.4] (<0.001)	10.5^*∗*^ [2.6–18.4] (0.009)
SSP	N/A	N/A	0.1 [−0.4 to 0.6] (0.676)	1^*∗*^ [0.1–1.9] (0.038)	0.00 [−0.01 to 0.01] (0.814)	24.1^*∗*^ [13.6–34.6] (<0.001)	8.3^*∗*^ [0.4–16.3] (0.039)
ISP	N/A	N/A	1^*∗*^ [0.5–1.5] (<0.001)	4.8^*∗*^ [3.8–5.7] (<0.001)	0.01^*∗*^ [0–0.02] (0.011)	0.2 [−10.3 to 10.7] (0.972)	5.5 [−2.4 to 13.4] (0.175)
SSP + SSC_sup_	N/A	N/A	0.9^*∗*^ [0.4–1.4] (0.001)	2.1^*∗*^ [1.2–3.1] (<0.001)	0.02^*∗*^ [0.01–0.03] (0.001)	46.9^*∗*^ [36.4–57.4] (<0.001)	22.6^*∗*^ [14.7–30.5] (<0.001)
SSP + ISP	N/A	N/A	1.1^*∗*^ [0.6–1.6] (<0.001)	5.3^*∗*^ [4.4–6.2] (<0.001)	0.02^*∗*^ [0.01–0.03] (0.001)	31.1^*∗*^ [20.6–41.5] (<0.001)	18^*∗*^ [10.1–26] (<0.001)
SSP + SSC_sup_+ ISP	N/A	N/A	1.5^*∗*^ [1–2] (<0.001)	4.3^*∗*^ [3.4–5.3] (<0.001)	0.03^*∗*^ [0.02–0.04] (<0.001)	64.1^*∗*^ [53.6–74.6] (<0.001)	23.5^*∗*^ [15.6–31.4] (<0.001)
Model fit							
Marginal R^2^ (conditional R^2^)	0.627 (0.623)	0.676 (0.673)	0.854 (0.848)	0.711 (0.7)	0.827 (0.821)	0.98 (0.98)	0.952 (0.95)
ICC_1_	0.000	0.008	0.263	0.060	0.000	0.345	0.385
ICC_2_	0.246	0.140	0.007	0.034	N/A	0.154	0.111
ICC_3_	0.008	0.032	0.810	0.326	0.823	0.487	0.527

^*∗*^*p* < 0.05. Excluded fixed effects variables from the stepwise linear regression are not available (N/A) for the linear mixed effects model. ICC_1_–intraclass correlation nested in sex; ICC_2_–intraclass correlation nested in sex on weight bearing; ICC_3_–intarclass correlation nested in specimens; CSA–critical shoulder angle; SSC_sup_–rotator cuff (RC) tear of the superior portion of the subscapularis muscle; SSP–RC tear of the supraspinatus muscle; ISP–RC tear of the infraspinatus muscle; SSP + SSC_sup_–RC tear of SSP + SSC_sup_; SSP + ISP–RC tear of SSP + ISP; SSP + SSC_sup_ + ISP–RC tear of SSP+SSC_sup_ + ISP

**Table 2:** Fixed effects estimate [confidence interval] (*p*-values) of the linear mixed effects model for the muscle forces at 30° abduction of the deltoid (anterior (DELT_ant_), middle (DELT_mid_), and posterior (DELT_post_)), the rotator cuff (RC; superior portion (SSC_sup_) and inferior portion of the subscapularis (SSC_inf_), supraspinatus (SSP), infraspinatus (ISP), teres minor (TMin)), pectoralis major (PECMaj), and latissimus dorsi (LAT) muscles.

Model parameters	Outcome–muscle force									
	DELT_ant_	DELT_mid_	DELT_post_	SSC_sup_	SSC_inf_	SSP	ISP	TMin	PECMaj	LAT
Fixed effects										
Intercept	49.2^*∗*^ [30.6–67.7] (<0.001)	70.9^*∗*^ [48.0–93.7] (<0.001)	11.0^*∗*^ [4.9–17.1] (<0.001)	30.0^*∗*^ [17.5–42.5] (<0.001)	16.7 [−2.7 to 36.2] (0.091)	22.6^*∗*^ [10.4–34.7] (<0.001)	23.7^*∗*^ [18.7–29.7] (<0.001)	18.3^*∗*^ [8.0–28.5] (<0.001)	8.1^*∗*^ [4.2–12.0] (<0.001)	−1.7 [−5.2 to 1.8] (0.347)
Weight-bearing task	32.5^*∗*^ [26.4–38.6] (<0.001)	83.4^*∗*^ [80.5–86.3] (<0.001)	0.7 [−0.6 to 2.0] (0.287)	10.1^*∗*^ [7.8–12.5] (<0.001)	24.9^*∗*^ [18.2–31.6] (<0.001)	6.0^*∗*^ [3.5–8.5] (<0.001)	2.5^*∗*^ [1.7–3.3] (<0.001)	7.7^*∗*^ [5.5–9.8] (<0.001)	−0.4 [−1.3 to 0.5] (0.391)	0.2 [−0.1 to 0.4] (0.272)
CSA	−0.4 [−0.8 to 0.1] (0.083)	0.7^*∗*^ [0.1–1.3] (0.028)	−0.1 [−0.2 to 0.0] (0.076)	0.1 [−0.2 to 0.4] (0.491)	0.0 [−0.4 to 0.5] (0.877)	0.1 [−0.2 to 0.4] (0.402)	−0.1 [−0.2 to 0.0] (0.104)	−0.1 [−0.4 to 0.2] (0.392)	0.0 [0–0.1] (0.427)	0.2^*∗*^ [0.1–0.2] (<0.001)
SSC_sup_	3.8 [−2.3 to 9.8] (0.218)	5.9 [−3.0 to 14.7] (0.192)	1.5 [−0.3 to 3.3] (0.093)	−44.7 ^*∗*^ [−49.5 to −39.8] (<0.001)	40.6^*∗*^ [33.8–47.4] (<0.001)	8.6^*∗*^ [4.2–12.9] (<0.001)	0.3 [−1.7 to 2.3] (0.772)	0.3 [−3.5 to 4.1] (0.877)	−0.7 [−1.8 to 0.4] (0.2)	0.1 [−0.7 to 0.9] (0.762)
SSP	4.9 [−1.2 to 10.9] (0.114)	30^*∗*^ [21.1–38.8] (<0.001)	1.3 [−0.5 to 3.1] (0.165)	8.4^*∗*^ [3.6–13.3] (0.001)	8.4^*∗*^ [1.6–15.2] (0.015)	−30.0 ^*∗*^ [−34.3 to −25.6] (<0.001)	4.4^*∗*^ [2.4–6.5] (<0.001)	4.4^*∗*^ [0.6–8.2] (0.024)	−0.1 [−1.1 to 1] (0.924)	0.3 [−0.5 to 1.2] (0.414)
ISP	6.9^*∗*^ [0.9–13] (0.025)	7.6 [−1.2 to 16.5] (0.09)	−1.4 [−3.2 to 0.4] (0.127)	−1.8 [−6.6 to 3.1] (0.468)	−1.8 [−8.6 to 5.0] (0.605)	4.3 [−0.1–8.6] (0.056)	−22.4 ^*∗*^ [−24.4 to −20.3] (<0.001)	11.5^*∗*^ [7.6–15.3] (<0.001)	−0.3 [−1.4 to 0.8] (0.556)	−0.1 [−0.9 to 0.7] (0.834)
SSP + SSC_sup_	15.2^*∗*^ [9.2–21.3] (<0.001)	42.1^*∗*^ [33.3–51.0] (<0.001)	0.7 [−1.1 to 2.5] (0.439)	−44.7 ^*∗*^ [−49.5 to −39.8] (<0.001)	58.3^*∗*^ [51.5–65.1] (<0.001)	−29.9 ^*∗*^ [−34.2 to −25.5] (<0.001)	5.2^*∗*^ [3.1–7.2] (<0.001)	5.2^*∗*^ [1.3–9.0] (0.009)	−0.6 [−1.7 to 0.5] (0.292)	−0.2 [−1.0 to 0.7] (0.712)
SSP + ISP	15.4^*∗*^ [9.3–21.5] (<0.001)	41.1^*∗*^ [32.3–50.0] (<0.001)	−0.9 [−2.7 to 0.9] (0.306)	9.2^*∗*^ [4.4–14.0] (<0.001)	9.2^*∗*^ [2.4–16] (0.008)	−31.8 ^*∗*^ [−36.1 to −27.4] (<0.001)	−22.4 ^*∗*^ [−24.4 to −20.3] (<0.001)	22.2^*∗*^ [18.4–26.1] (<0.001)	0.0 [−1.1 to 1.1] (0.974)	−0.2 [−1.0 to 0.6] (0.633)
SSP + SSC_sup_+ ISP	29.3^*∗*^ [23.3–35.4] (<0.001)	53.4^*∗*^ [44.5–62.2] (<0.001)	−0.8 [−2.7 to 1.0] (0.356)	−44.7 ^*∗*^ [−49.5 to −39.8] (<0.001)	61.6^*∗*^ [54.8–68.4] (<0.001)	−30.8 ^*∗*^ [−35.1 to −26.4] (<0.001)	−22.4 ^*∗*^ [−24.4 to −20.3] (<0.001)	25.2^*∗*^ [21.3–29.0] (<0.001)	0.8 [−0.3 to 1.9] (0.147)	−0.1 [−1.0 to 0.7] (0.728)
Model fit										
Marginal *R*^2^ (conditional *R*^2^)	0.879 (0.874)	0.945 (0.943)	0.589 (0.573)	0.884 (0.880)	0.877 (0.872)	0.832 (0.825)	0.909 (0.905)	0.729 (0.719)	0.632 (0.618)	0.606 (0.590)
ICC_1_	0.111	0.000	0.423	0.026	0.075	0.139	0.000	0.029	0.288	0.631
ICC_2_	0.068	0.000	0.029	0.016	0.078	0.023	0.007	0.023	0.038	0.000
ICC_3_	0.363	0.096	0.423	0.077	0.255	0.179	0.023	0.161	0.512	0.631

^*∗*^*p* < 0.05. Excluded fixed effects variables from the stepwise linear regression are not available (N/A) for the linear mixed effects model. ICC_1_–intraclass correlation nested in sex; ICC_2_–intraclass correlation nested in sex on weight bearing; ICC_3_–intarclass correlation nested in specimens; CSA–critical shoulder angle; SSC_sup_–rotator cuff (RC) tear of the SSC_sup_; SSP–RC tear of the SSP; ISP–RC tear of the ISP; SSP + SSC_sup_–RC tear of SSP + SSC_sup_; SSP + ISP–RC tear of SSP + ISP; SSP+SSC_sup_ + ISP–RC tear of SSP + SSC_sup_ + ISP.

**Table 3:** Fixed effects estimates [confidence interval] (*p*-values) of the linear mixed effects model summary for the time-averaged muscle forces of the deltoid (anterior (DELT_ant_), middle (DELT_mid_), and posterior (DELT_post_)), the rotator cuff (RC; superior portion (SSC_sup_) and inferior portion of the subscapularis (SSC_inf_), supraspinatus (SSP), infraspinatus (ISP), teres minor (TMin)), pectoralis major (PECMaj), and latissimus dorsi (LAT) muscles.

Model parameters	Outcome–muscle force									
	DELT_ant_	DELT_mid_	DELT_post_	SSC_sup_	SSC_inf_	SSP	ISP	TMin	PECMaj	LAT
Fixed effects										
Intercept	25.3^*∗*^ [15.8–34.8] (<0.001)	36.4^*∗*^ [26.9–45.8] (<0.001)	9.2^*∗*^ [5.3–13.1] (<0.001)	23.5^*∗*^ [17.3–29.8] (<0.001)	15.9^*∗*^ [5.1–26.7] (0.004)	17.3^*∗*^ [11.2–23.3] (<0.001)	21.9^*∗*^ [19.4–24.5] (<0.001)	19.6^*∗*^ [13.5–25.7] (<0.001)	8.2^*∗*^ [5.0–11.3] (<0.001)	3.9^*∗*^ [0.18–7.6] (0.040)
Weight-bearing task	16.3^*∗*^ [12.9–19.8] (<0.001)	41.4^*∗*^ [40.2–42.5] (<0.001)	−0.4 [−1 to 0.2] (0.198)	4.6^*∗*^ [3.2–5.9] (<0.001)	11.8^*∗*^ [8.4–15.1] (<0.001)	2.5^*∗*^ [1.3–3.8] (<0.001)	1.1^*∗*^ [0.5–1.7] (<0.001)	3.6^*∗*^ [1.9–5.3] (<0.001)	−0.5 ^*∗*^ [−0.9 to −0.1] (0.009)	−0.1 [−0.4 to 0.2] (0.498)
CSA	−0.1 [−0.3 to 0.1] (0.181)	0.5^*∗*^ [0.2–0.7] (<0.001)	0.0 [−0.1 to 0.0] (0.498)	0.1 [−0.1 to 0.2] (0.507)	0.0 [−0.2 to 0.3] (0.718)	0.0 [−0.1 to 0.2] (0.51)	−0.1 ^*∗*^ [−0.2 to 0.0] (0.013)	−0.1 [−0.3 to 0.0] (0.184)	0.0 [0–0.1] (0.699)	0.1^*∗*^ [0–0.1] (0.005)
SSC_sup_	3.9^*∗*^ [1.6–6.1] (0.001)	5.1^*∗*^ [1.6–8.6] (0.005)	−1.0 ^*∗*^ [−1.9 to 0] (0.047)	−30.3 ^*∗*^ [−32.7 to −28] (<0.001)	26.2^*∗*^ [22.6–29.7] (<0.001)	4.0^*∗*^ [2.0–6.0] (<0.001)	0.9 [−0.1 to 2] (0.072)	0.9 [−1.3 to 3.1] (0.398)	−1.1 ^*∗*^ [−1.8 to −0.3] (0.004)	0.6^*∗*^ [0.1–1.1] (0.015)
SSP	4.1^*∗*^ [1.9–6.4] (<0.001)	16.4^*∗*^ [12.9–19.9] (<0.001)	0.0 [−1 to 0.9] (0.957)	3.8^*∗*^ [1.4–6.1] (0.002)	3.8^*∗*^ [0.2–7.3] (0.038)	−18.0 ^*∗*^ [−20.0 to −16.1] (<0.001)	2.0^*∗*^ [0.9–3] (<0.001)	2.0 [−0.2 to 4.2] (0.081)	−0.6 [−1.3 to 0.1] (0.107)	0.1 [−0.4 to 0.6] (0.702)
ISP	6.9^*∗*^ [4.6–9.1] (<0.001)	9.1^*∗*^ [5.5–12.6] (<0.001)	4.6^*∗*^ [3.7–5.6] (<0.001)	−1.6 [−3.9 to 0.8] (0.191)	−1.6 [−5.1 to 2.0] (0.39)	2.6^*∗*^ [0.7–4.6] (0.009)	−20.1 ^*∗*^ [−21.1 to −19.1] (<0.001)	6.6^*∗*^ [4.4–8.8] (<0.001)	3.5^*∗*^ [2.8–4.2] (<0.001)	−0.1 [−0.6 to 0.4] (0.704)
SSP + SSC_sup_	11.2^*∗*^ [8.9–13.4] (<0.001)	27.2^*∗*^ [23.7–30.7] (<0.001)	−1.4 ^*∗*^ [−2.3 to −0.4] (0.004)	−30.3 ^*∗*^ [−32.7 to −28] (<0.001)	35.4^*∗*^ [31.8–38.9] (<0.001)	−18.0 ^*∗*^ [−19.9 to −16.0] (<0.001)	2.7^*∗*^ [1.7–3.7] (<0.001)	2.7^*∗*^ [0.5–4.9] (0.016)	−1.1 ^*∗*^ [−1.8 to −0.3] (0.005)	0.3 [−0.2 to 0.8] (0.173)
SSP + ISP	12.4^*∗*^ [10.1–14.6] (<0.001)	27.7^*∗*^ [24.1–31.2] (<0.001)	3.2^*∗*^ [2.3–4.2] (<0.001)	3.8^*∗*^ [1.5–6.2] (0.001)	3.8^*∗*^ [0.3–7.4] (0.035)	−18.7 ^*∗*^ [−20.7 to −16.8] (<0.001)	−20.1 ^*∗*^ [−21.1 to −19.1] (<0.001)	12.7^*∗*^ [10.5–14.9] (<0.001)	2.0^*∗*^ [1.3 to 2.8] (<0.001)	−0.2 [−0.7 to 0.3] (0.437)
SSP + SSC_sup_+ ISP	17.7^*∗*^ [15.5–20] (<0.001)	34.2^*∗*^ [30.7–37.7] (<0.001)	−2.0 ^*∗*^ [−2.9 to −1.0] (<0.001)	−30.3 ^*∗*^ [−32.7 to −28.0] (<0.001)	33.5^*∗*^ [29.9–37.0] (<0.001)	−18.1 ^*∗*^ [−20.1 to −16.1] (<0.001)	−20.1 ^*∗*^ [−21.1 to −19.1] (<0.001)	14.4^*∗*^ [12.2–16.6] (<0.001)	−0.5 [−1.2 to 0.3] (0.2)	0.0 [−0.5 to 0.5] (0.862)
Model fit										
Marginal *R*^2^ (conditional *R*^2^)	0.939 (0.936)	0.965 (0.964)	0.779 (0.771)	0.930 (0.927)	0.89 (0.885)	0.891 (0.887)	0.967 (0.965)	0.729 (0.718)	0.791 (0.783)	0.877 (0.872)
ICC_1_	0.383	0.000	0.528	0.047	0.110	0.259	0.006	0.075	0.374	0.812
ICC_2_	0.091	0.000	0.017	0.026	0.061	0.029	0.030	0.050	0.006	0.004
ICC_3_	0.599	0.188	0.581	0.141	0.352	0.306	0.027	0.193	0.678	0.865

^*∗*^*p* < 0.05. Excluded fixed effects variables from the stepwise linear regression are not available (N/A) for the linear mixed effects model. ICC_1_–intraclass correlation nested in sex; ICC_2_–intraclass correlation nested in sex on weight bearing; ICC_3_–intarclass correlation nested in specimens; CSA–critical shoulder angle; SSC_sup_–rotator cuff (RC) tear of the SSC_sup_; SSP–RC tear of the SSP; ISP–RC tear of the ISP; SSP + SSC_sup_–RC tear of SSP + SSC_sup_; SSP + ISP–RC tear of SSP+ISP; SSP + SSC_sup_ + ISP–RC tear of SSP + SSC_sup_ + ISP

## Data Availability

Additional data are available in the Supplementary Material, and the descriptive statistics used to support the results of this study are deposited in the “Descriptive statistics supplement: Influence of shoulder morphology and rotator cuff tear type on load-induced glenohumeral biomechanics—a Sawbone simulator study repository” (https://doi.org/10.5281/zenodo.10663334) [27].

## Supplementary Materials

Supplementary Materials Supplementary Descriptive Statistics: Mean results of each outcome variable (glenohumeral translation, center of force, instability ratio, magnitude of the glenohumeral joint reaction force, and muscle forces) grouped by small, healthy, and large critical shoulder angle (CSA), weight bearing, and an overall mean in each weight-bearing level along with first and third quartiles are provided (Zenodo: [27]). Supplement Mean Trajectory: Box plots of anterior glenohumeral translations and graphs of trajectories of outcomes (glenohumeral translation, center of force, instability ratio, magnitude of glenohumeral joint reaction force, and muscle forces) grouped by rotator cuff tear and weight-bearing load are provided.
