# Supplementary material for: Influence of Critical Shoulder Angle and Rotator Cuff Tear Type on Load-Induced Glenohumeral Biomechanics: A Sawbone Simulator Study
Source: Appl Bionics Biomech. 2024 Jul 2;2024:4624007. doi: 10.1155/2024/4624007 (PMC11233187; doi:10.1155/2024/4624007)
Supplement: Supplementary Materials — Supplementary Descriptive Statistics: Mean results of each outcome variable (glenohumeral translation, center of force, instability ratio, magnitude of the glenohumeral joint reaction force, and muscle forces) grouped by small, healthy, and large critical shoulder angle (CSA), weight bearing, and an overall mean in each weight-bearing level along with first and third quartiles are provided (Zenodo: [27]). Supplement Mean Trajectory: Box plots of anterior glenohumeral translations and graphs of trajectories of outcomes (glenohumeral translation, center of force, instability ratio, magnitude of glenohumeral joint reaction force, and muscle forces) grouped by rotator cuff tear and weight-bearing load are provided. [file 4624007.f1.docx]

# Supplementary Materials


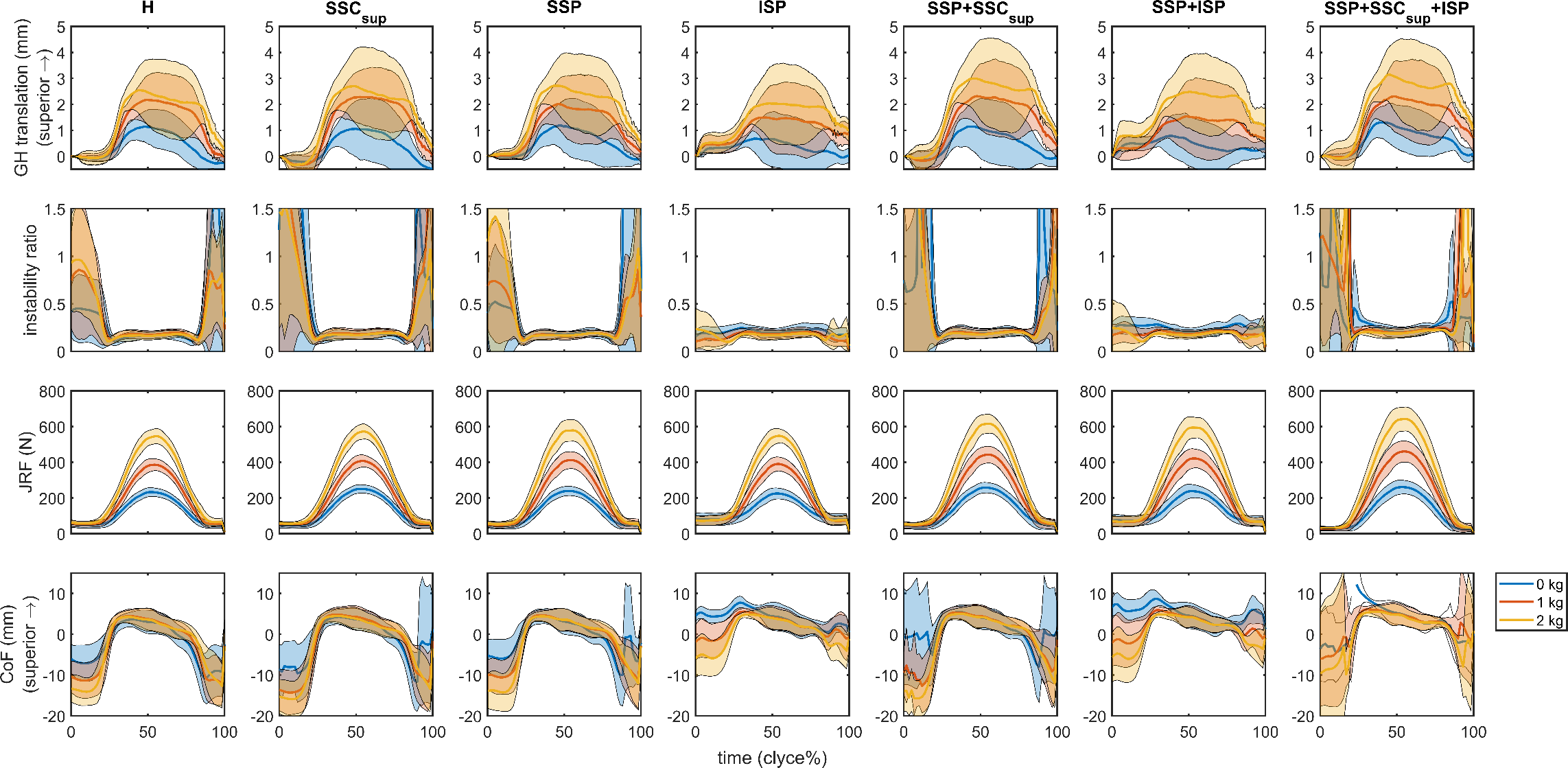


Figure 1A: Trajectories of glenohumeral (GH) translations, instability ratio, joint reaction forces (JRF), and center of force (CoF) grouped by tear type and load; the column represents different RC tear types and each row represents a different muscle. Of note are the instability ratios at the beginning and end of the movement cycle. They are significantly increased as the humeral head is centered at the beginning and the joint compressive pressure is relaxed at the end.


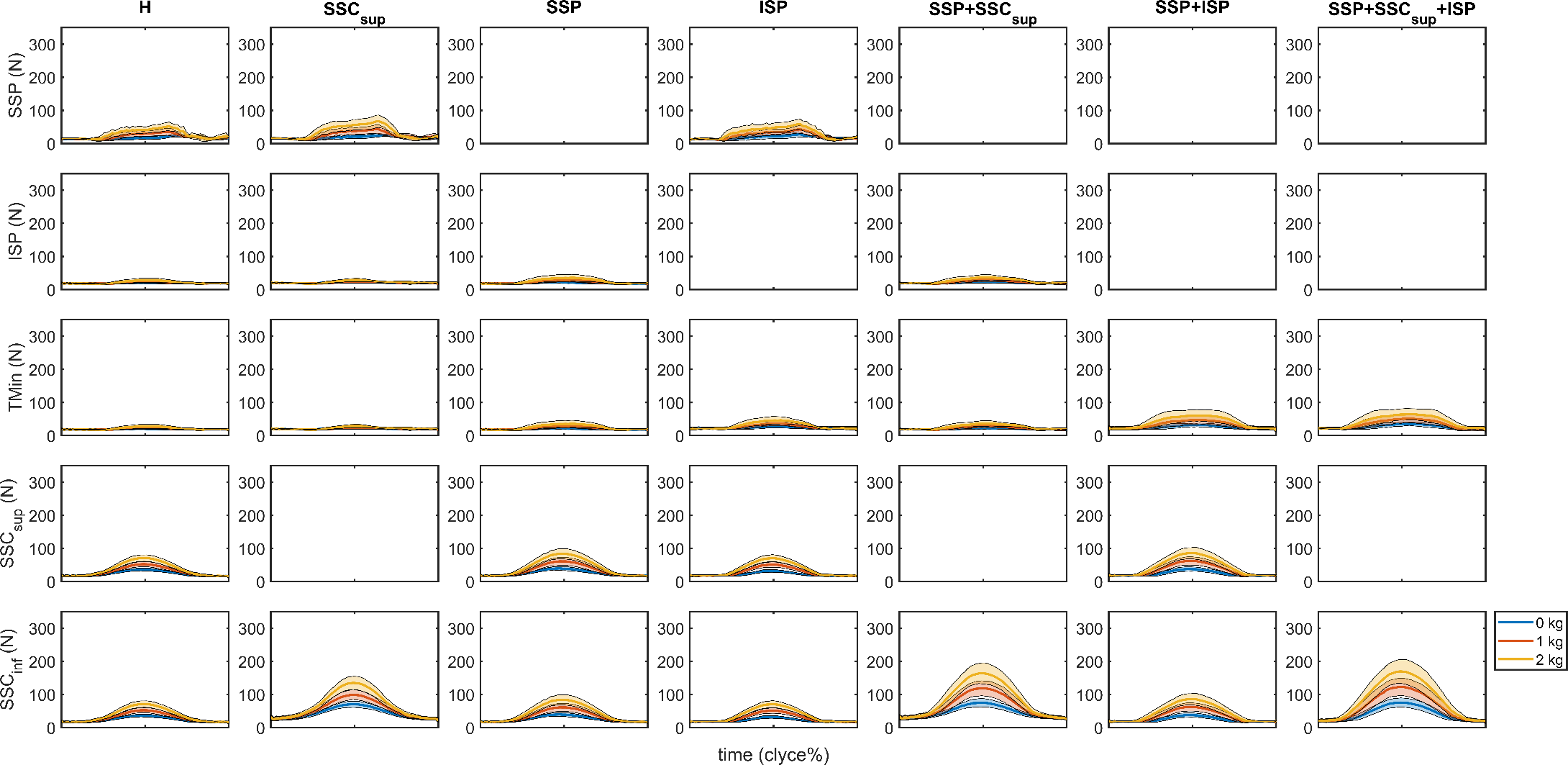


Figure 2A: Trajectories of rotator cuff (RC) Muscle forces; the column represents different RC tear types and each row represents a different muscle. Legend: *H – intact RC, SSP – supraspinatus; SSC_sup_ –superior subscapularis portion; SSC_inf_ – inferior subscapularis; ISP – infraspinatus; TMin – teres minor.*


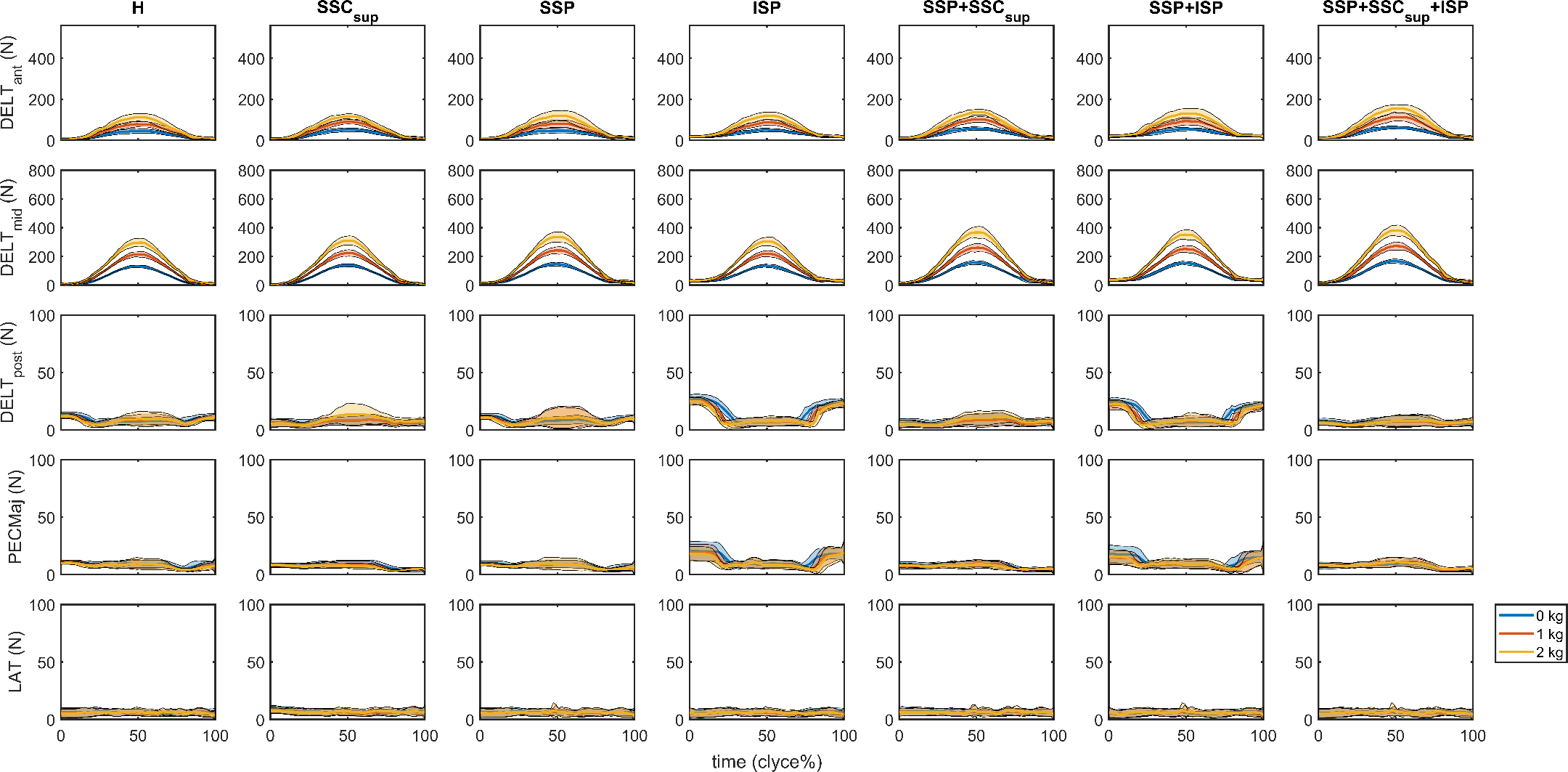


Figure 3A: Trajectories of glenohumeral Muscle forces; the column represents different RC tear types and each row represents a different muscle. Legend: *H – intact rotator cuff; DELT_ant_ –anterior deltoid portion; DELT_mid_ –middle deltoid portion; DELT_post_ posterior deltoid portion; SSP – supraspinatus; SSC_sup_ –superior subscapularis portion; ISP – infraspinatus; PECMaj – pectoralis major; LAT – latissimus dorsi.*


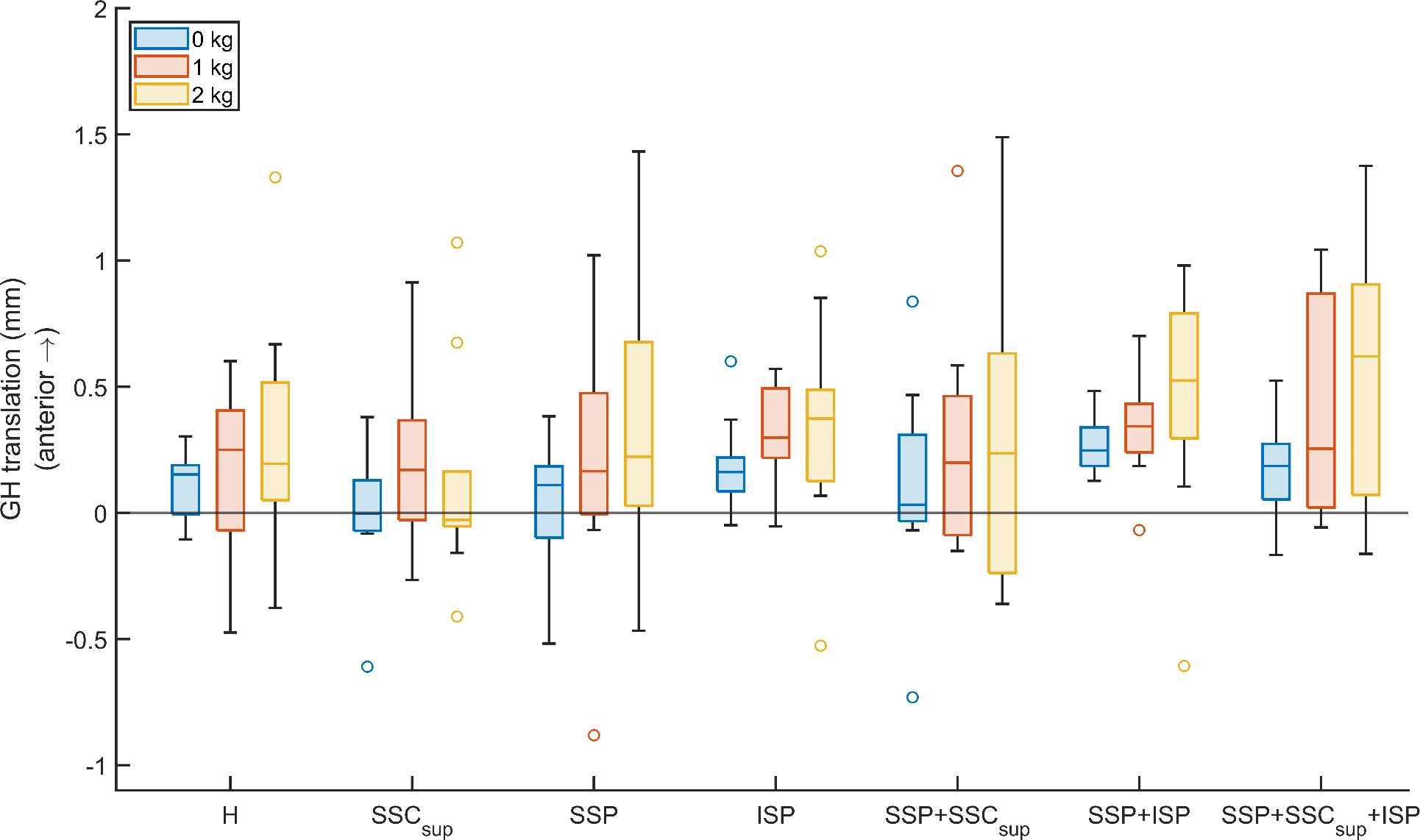


Figure 3A: Boxplot of the glenohumeral (GH) anterior translation, at 30° abduction grouped by rotator cuff (RC) tear type and weight bearing loads (0 kg, 1 kg and 2 kg). Legend: H – intact RC; rotator cuff tears: SSC_sup_ –superior portion of the subscapularis muscle; SSP –supraspinatus muscle; ISP –infraspinatus muscle.
